# Use of Psychotropic “Pro Re Nata” Medications by Patients Attending the Outpatient Clinic in a Hospital: A Qualitative Exploration

**DOI:** 10.3389/fmed.2021.617147

**Published:** 2021-07-23

**Authors:** Kingston Rajiah, Mari Kannan Maharajan, Hemawathi Ramaya, Wan Nur Asyiken Wan Ab Rahman

**Affiliations:** ^1^Department of Pharmacy Practice, School of Pharmacy, International Medical University, Kuala Lumpur, Malaysia; ^2^School of Postgraduate Studies, International Medical University, Kuala Lumpur, Malaysia; ^3^Hospital Kajang, Kajang, Malaysia

**Keywords:** self-medication, self-management, decision-making, patient autonomy, psychotropic medicine, medication safety

## Abstract

**Introduction:** Administration of psychotropic *pro re nata* (PRN) medications is influenced by diverse factors such as legal use of PRN medications, the attitude of patients, personal bias, and stigma toward such medication use. While PRN prescriptions increase the efficiency of care and encourage patients to participate in self-care, the use of psychotropic PRN medications by outpatients has raised concerns about its risks of harm, especially for the outpatients. This study explored the use of psychotropic PRN medications by patients attending the outpatient clinic in a hospital.

**Methods:** Qualitative in-depth interviews were conducted. Purposeful sampling was done to achieve cases with enriched information. Participants were chosen regardless of their ethnicity and were selected using the database and patient records in the clinic. Patients 18 years of age prescribed PRN psychotropic medications attending outpatient clinics in a hospital were included. Vulnerable patients (e.g., pregnant ladies, prisoners, cognitively impaired individual, AIDS/HIV subjects, and terminally ill subjects) were excluded.

**Results:** This study revealed the patients' perspectives and experiences on self-management of psychotropic PRN medications. The themes that emerged were clustered as education and background, knowledge on psychotropic medications, frequency of medication intake, underuse of medication, the overdose of medication, side effects concern, source of information, and personal experience.

**Conclusions:** Patients' understanding of medication, inappropriate medication use, cues to action, and use of alternatives are the factors that affected the self-management of psychotropic PRN medications by the patients.

## Introduction

Psychotropic medications are the mainstay in the management of mental health (1). “Pro re nata” (PRN) psychotropic medications are frequently prescribed in mental health management for various reasons such as insomnia, agitation, in occasions of violence, symptom distress, and aggression (2). Psychotropic PRN medications are utilized in inpatient units, general medical units, and outpatient clinics in hospitals (3). Proper use of psychotropic PRN medications goes beyond the pharmacology and indications of specific therapeutic agents (4). It involves legal use of PRN medications, the attitude of patients, personal bias, and stigma toward such medication use. PRN literally means “according to the circumstances it may require” (5). Psychotropic PRN medication management needs assessment and judgment of the mental health condition of patients and appropriate medications (6); and psychiatrists, who have knowledge on mental health conditions and the physiological responses of psychotropic medications, are involved in the decision making (5, 6). Due to its complexity in psychotropic PRN medications, healthcare professionals provide close supervision to the inpatients. However, no such monitoring is possible for the outpatients, where medical advice is required to patients and patients have to take ownership in managing their conditions. In such cases, the role of healthcare professionals in medication counseling on the indications, complications, and possible interactions of psychotropic PRN medications is very important (4, 7).

Self-management is self-directed, which encompasses various approaches that an individual may use on his/her own, without any guidance by a professional, in managing his/her symptoms (8). Self-management of medications by patients depends on their motivation, values and beliefs, trustworthiness with their doctors, and their confidence in medicines prescribed to them (9). Previous studies on self-management of psychotropic PRN medications had been conducted in inpatients (8, 10–15). These studies revealed that inpatients had a minimal understanding of medications and do not support to use alternatives. The patients could not make the right decisions if the information given to them was unclear. This is also raised concerns among researchers and policymakers on the effectiveness and empowerment of patients in self-management. Despite the widespread use of psychotropic PRN medications, there is no guideline on the use of psychotropic PRN medications for outpatients (13, 16, 17). While self-management of psychotropic medications among inpatients has significant apprehensions, self-management of psychotropic PRN medications by patients has not been explored. There are research gaps regarding the patient safety concerns among the outpatients using psychotropic PRN medications. Systematic reviews on psychotropic PRN medications have suggested that qualitative approach among mental health patients is needed, which might address the medication-related problems (4, 18, 19). Hence, to focus on these issues, there is a need for a qualitative study among the patients using psychotropic PRN medications in outpatient settings. Therefore, this study is designed to explore the use of psychotropic PRN medications by patients attending the outpatient clinic in a hospital.

## Methods

### Ethical Approval

The study was approved by the IMU Joint Committee of Research and Ethics [MPP 1/2019 (07)].

### Study Setting

The study was conducted in the Psychiatry Department in Hospital Kajang, Malaysia, one of the leading centers for psychiatry in this country. This is a tertiary care hospital serving many psychiatric outpatients.

### Study Participants

In this study, participants were selected from the psychiatry outpatient clinic between September 2019 and January 2020. Purposeful sampling was done to achieve cases with enriched information. Participants were chosen regardless of their ethnicity and were selected using the database and patient records in the clinic. Patients 18 years of age prescribed PRN psychotropic medications attending outpatient clinics at Hospital Kajang were included. Vulnerable patients (e.g., pregnant ladies, prisoners, cognitively impaired individual, AIDS/HIV subjects, and terminally ill subjects) were excluded. The information obtained was kept confidential. Participants were informed regarding the objectives of the study using a patient information sheet. Before beginning the interview, the researcher briefed each participant on the interview process. Written consent was obtained preceding data collection. A total of 12 patients were approached for interviews; nevertheless, a saturation point was reached at the 10th interview, and no new information was gained from the succeeding interviews, achieving the required sample size for phenomenological approaches (20).

### Study Tool

Following a vast literature review, researchers created a semi-structured interview guide for data collection. After discussion with experts, the guide was modified subsequently. Open-ended questions were preferred to provide interviewees with a full opportunity to convey their opinions and help in obtaining a greater understanding of issues (21, 22). A pilot interview was conducted with two patients. The probes found during the pilot interview were included in the interview guide preceding its final use. The data collected during the pilot interview were not included in the results.

### Interview Process

In-depth interviews were conducted for gathering information in view of the sociocultural perspective (22). All interviews were performed by a researcher who is trained in qualitative methods and interview processes. Interviews were performed in English, as most of the participants were comfortable with it. The national language (Malay) was also used while using probing questions to get a thorough understanding of issues. Approximately 25–40 min was taken to complete each interview. Every interview was audio recorded by the researchers. Field notes were taken during data collection. Participants' approval was obtained for each transcribed interview verbatim.

### Data Analysis

Thematic content analysis was done for all transcribed interviews, and emerging themes were identified by analyzing the transcripts (23). This analysis followed six steps. The first step was becoming familiar with the data. All the transcripts were carefully read many times to get a clear sense. At this stage, the researchers made notes and wrote down the initial impressions. The second step was generating initial codes. The researchers organized the data in a profound way. Coding reduced the large data into small meaningful data. The third step was identifying themes. The researchers examined the codes and some of the codes that fitted together into one or more themes. The codes were organized into preliminary themes that are specific to this study. Step 4 was the review of themes. In this step, the preliminary themes were reviewed, modified, and developed. Step 5 was defining the themes. In this step, the themes were refined. Subthemes were refined and ensured how they relate and interact to the main themes. Step 6 was a write-up. The integrity of the data was maintained by identifying theme clusters and developing the themes. Finally, an experienced qualitative researcher independently reviewed the themes identified by the researchers.

### Study Trustworthiness

The trustworthiness was recognized at various phases of this study (24). A reflective journal was used while taking field notes to reduce researcher bias (25). Participants' characteristics were referred to address the transferability of the data. Completeness and credibility of the content were ensured by establishing the sample size based on saturation. In this study, the unit of analysis was chosen as sentences instead of letter/words to give an appropriate meaning to the text.

## Results

The summary of quatations from the focus group dicussions is mentioned in Table 1.

**Table 1:** Data matrix of thematic analysis.

**Themes emerged**	**Theme cluster**
Patients' understanding	Education and background
	Knowledge of psychotropic medications
Medication safety	Frequency of medication intake
	Underuse of medication
	Overdose of medication
	Side effects concern
Cues to action	Source of information
	Personal experience
Alternatives	Alternative medicines and therapies

### Theme 1: Patients' Understanding

The results revealed that the participants' understanding of psychotropic PRN medication varied according to their education and background. There was a difference in patients' understanding according to the knowledge on psychotropic medications.

### Education and Background

Participants living in the rural region, who have low family income, and who have poor education have difficulty in understanding the medications.

“My home in kampong area la… My family also don't know about these medicines. So, I don't understand the medicine.” (P12)

“I don't know much about this. I stopped school because of poor family income. I don't know the name, but it makes me cool, it makes me calm down.” (P5)

### Knowledge of Psychotropic Medication

Participants who have knowledge of psychotropic medications were able to understand the type of medications they are taking and able to follow the instructions by the healthcare providers.

“I am taking escitalopram, sertraline and alprazolam for my depression and bipolar problem.” (P6)

“He mentions (referring to the doctor) if I need… if I need… this medicine is like… Hmmm… I mean it can help me sleep… and if I really need it, like I'm traumatic… if I really need it, otherwise no need to take.” (P4)

“Pharmacy also got told to take the medicine when necessary and if need only, don't take many doses. This medicine is strong… only when need…. if no, then no need.” (P11)

“I cannot take it regularly because it makes me addicted. Without this medicine I cannot live, something like that.” (P5)

### Theme 2: Medication Safety

The results revealed that the participants use the medications in inappropriate ways such as inaccurate frequency, underuse, overdose, and side effects of medications.

### Frequency of Medication Intake

Most of the participants take the medications on a daily basis, though the medications have been prescribed on PRN basis. The reasons quoted by the participants are inability to sleep and dependence.

“I take the medicine every day because without that I feel somewhat difficult …if I take every day definitely the medicine finish already… So, I keep buying.” (P8)

“I eat every day because I need if not, I cannot sleep. The doctor only gives 20, not enough. Until morning also, I cannot sleep… but I take only at night before sleep… but cannot sleep… Hmmm and not enough.” (P2)

### Underuse of Medication

Most of the patients do not take the medications as prescribed. They skip the medications when needed or take a lesser dose than what is prescribed for them.

“I take only half tablet… no I don't take more… I scared.” (Almost all the patients)

“The one Putrajaya gives me is big, this one is very small, and I take half only… the doctor tells to take half… the dose is very less… the tablet very small… I don't take that …it won't work I think.” (P7)

### Overdose of Medication

Some participants take more dose than what is prescribed. These patients do not take medications on a daily basis. They take medications on a PRN basis, but the dose is more than required. The reason quoted by the patients is to calm themselves rapidly.

“I want to calm down and I don't want to get admitted in the hospital… drips this one, that one, people come and visiting me. I don't want that to happen. So, I take more than what doctor said.” (P3)

“I want to be happy and calm down. But that time when I don't have, I eat the expired one. I know eat the expired one got many effects, but that doctor don't want to give so where I can find.” (P10)

### Side Effects Concern

Some participants are concerned about the side effects of the medications, which have led them to avoid the medications. The side effects quoted by the participants are palpitation, vomiting, dizziness, skin rash, irritation in the eye, weight gain, and addiction.

“I know it can give good sleep, rest, but has side effects also. It can increase the palpitation and everything…that's all I know. …it also can cause vomiting, dizziness, and I know it can cause eye reddish and skin rashes too. I know this from my sister who is a staff nurse.” (P9)

“It can cause addiction and I don't want to be addicted…haaa…that's why I don't want…I scared.” (P3)

“I heard; it can increase my weight. So, I am actually worried to take the tablet.” (P7)

### Theme 3: Cues to Action

Cues to action are the stimulus that initiates the participants' decision making to agree on recommended medications. The cues in this study were participants' personal experience (internal cue) and source of information (external cue).

### Source of Information

Most participants' decision making was based on the source of information. Participants obtained advice from doctors, at the same time obtained information from articles and online sources like the Google search engine.

“Doctors explain but I also study myself…every single medicine I take I study myself because of side effect. I also have colitis and hypertension and given Amlodipine 10 mg. But when I read an article from the year 2016, it states it can trigger colitis. So, I told the doctor I don't want Amlodipine.” (P7)

“So, the first time when I get this medicine from the pharmacist, I was surprised why I get this medicine. So, I said, hmmm now is internet world…so I google. Haaa from there I know. So, I don't want to depend on this medicine.” (P2)

### Personal Experience

Some participants' decision making was based on their personal experience. Some participants' experience had led to negative effect, as they experienced humiliation from families and friends. However, some participants' experience had led to positive effect, as they experienced relief after using the medications.

“I decided to stop this tablet as I experienced humiliation from my friends and sometimes by family members.” (P6)

“I decided to continue the medication as I feel much better and normal when I take this medication.” (P4)

### Theme 4: Alternatives

Participants had other alternative ways to manage their psychotropic conditions. The alternatives quoted by the participants are religious belief, acupuncture, homeopathy, traditional medicine, yoga, and sedative cough syrups.

### Alternative Medicines and Therapies

Most participants mentioned over-the-counter cough syrups, homeopathy medicines, and traditional medicines as their alternatives. Some patients mentioned acupuncture and yoga as alternative therapies.

“I am not crazy to take it every day… we must control ourselves la…we also have our religion… you know must do a lot of prayers. So, when I take, I pray to my god… whenever I try to sleep, I put some prayer song on my phone… then I try to sleep… like that, I try… if that also cannot, then I eat the medicine.” (P1)

“I tried many –many alternatives… ha.ha… ha (patient laughing)… alternative medication like everything… you know… acupuncture… I go to homeopathy right… I try yoga.” (P7)

“I got haaa you know….haaa ‘kampung' medicine…haa what…traditional medicine to help me.” (P4)

“I take cough syrups because it is sleepy. Hahahaha (patient laughing) …if my children got flu, I ask them not to finish the medicine. Take that medicine can make me sleepy, so I take it.” (P1)

## Discussion

Patients' perspectives and experiences on psychotropic PRN medications are essential to identify the methods to improve patient adherence and avoid medication safety issues. Healthcare outcomes of patients' mental health depend on how well the patients manage their medication appropriately. Inappropriate use of psychotropic PRN medications can lead to medication safety risks to the patients. It can affect the overall treatment outcomes, which may result in a burden for caregivers and society. This study revealed that patients' understanding of psychotropic PRN medication varies according to their education and background. Patients living in the rural region, who have low family income, and who have poor education had difficulty in understanding the psychotropic PRN medications. Also, patients' understanding of psychotropic PRN medications was better when their knowledge of the medications prescribed was high. Patients who have knowledge of psychotropic PRN medications were able to understand the type of medications they are taking and able to follow the instructions by the healthcare professionals. Hence, patients' knowledge of psychotropic PRN medications should be focused on, which may improve their understanding of psychotropic PRN medications. Studies have reported that patient education and counseling on medications may improve their knowledge of medications and enhance their understanding and self-management of medications (26, 27). Hence, patient education and counseling for psychotropic PRN medications should be given explicitly, which may ensure patients' self-management of medications.

Appropriate use of medication is a critical element for any therapeutic accomplishment and to avoid medication safety risks. This study revealed that the patients used their medications inappropriately in many ways, which is a prominent medication safety concern. Most patients took their medications on a daily basis, though their prescription was on a PRN basis. Some patients strictly used their medications on a PRN basis; however, they consumed more than the required dose. Patients also avoided their medications fully or consumed lesser than the prescribed doses intentionally. These patients mentioned that they tend to do this because these medications were affecting their sleep and causing side effects. Some patients wanted to calm themselves rapidly. Self-management of psychotropic PRN medications raises serious patient safety concern, as inappropriate medication use among mental health patients is prevalent. This shows that self-management of medications may not be appropriate for all the patients. The United Kingdom National Health Service has a scheme called self-administration of medication (SAM) for patients who self-manage their own prescribed medicines (28). According to this scheme, a multidisciplinary healthcare professional team should ensure the patients' capacity to be involved in the self-management of medications. Each patient must be individually assessed for their ability to self-use their prescribed medications. A systematic review of this scheme has reported that the scheme is effective in helping patients to increase their independence and regain control over some aspects of their care through improved knowledge of and greater compliance with their medication regimen (29). However, in Malaysia, there is no such scheme for the self-administration of medicines. Hence, the PRN medications affect the self-management of patients on psychotropic PRN medications with serious medication safety concerns.

Cues to action are the stimulus that initiates the participants' decision making to agree on recommended medications. The cues in this study were participants' personal experience (internal cue) and source of information (external cue). In this study, patients' decision making was based on the source of information and personal experiences. External cues were healthcare professionals and technologies, whereas internal cue was personal experience. Patient autonomy allows healthcare professionals to educate the patients; however, the decision can be made by patients. Studies have supported patient autonomy and allow them to make decisions regarding a specific medical intervention or treatment (30, 31). Patient well-being and patient autonomy are linked together. Patient autonomy should not be taken as “patient control,” but it is the responsibility of the healthcare professionals to educate and talk about the concerns on patient well-being (32). This may certainly help in the self-management of psychotropic PRN medications by patients.

Patients have many alternative ways to manage their psychotropic conditions. In this study, the patients considered various alternatives ways such as religious belief, acupuncture, homeopathy, traditional medicine, yoga, and sedative cough syrups. Patients tend to use alternatives, as they believe in a holistic approach to health and well-being (33). Health belief is interlinked with the body, mind, and spirit in health (34). Patients look for relief of symptoms from their personal experiences and hence look for alternative healthcare (33). The decision by the patients to use alternatives is situation dependent. It may be influenced by others who might not have similar medical conditions. Hence, healthcare professionals should be providing personalized care to help patients in self-management of psychotropic PRN medications and to educate them on medication safety concerns.

In Malaysia, the Ministry of Health has taken various measures to ensure the medication safety and use of consumers. As per Pharmaceutical Services Division report, medicine consumers in Malaysia are satisfied with the labeling of medicines, and there is no difficulty in reading the labels. However, consumers have difficulty in identifying medicines by their generic name. Consumers' information-seeking behavior has moved up from consulting the healthcare providers to accessing technologies. Though nationwide programs like “Know Your Medicines” and “My Health for life” are prevalent, participation is still low (35, 36). Despite all these measures by the Ministry of Health, there is a need for a self-medication management scheme in Malaysia for psychotropic medications, especially PRN medications. This may facilitate the patient's responsibility in managing their own psychotropic PRN medications. This scheme can consist of various stages and the use of psychotropic PRN medications, in which the patients and healthcare professionals can be active participants. A self-reporting scale can be incorporated inside the scheme, which can be used by the healthcare professionals and the patients to determine the patient's knowledge, and understanding and safe medication use.

## Limitations

This study had some limitations. This study was conducted at an outpatient clinic setting in a hospital, which may have influenced patients' opinions and perspectives. This study may not signify the views of those who defaulted to the outpatient visits.

## Conclusions

This study revealed the patients' perspectives and experiences on self-management of psychotropic PRN medications. Patients' understanding of medication, medication safety, cues to action, and use of alternatives are the factors that affected the self-management of psychotropic PRN medications by the patients. A quantitative investigation is needed to determine and evaluate the associated factors.

## Data Availability Statement

The original contributions presented in the study are included in the article/supplementary material, further inquiries can be directed to the corresponding author/s.

## Ethics Statement

The studies involving human participants were reviewed and approved by IMU Joint Committee of Research and Ethics [MPP 1/2019 (07)]. The patients/participants provided their written informed consent to participate in this study.

## Author Contributions

KR and HR: conceptualization. KR: methodology, writing—review and editing, project administration, and funding acquisition. MM: validation, resources, and visualization. HR: formal analysis, investigation, and writing—original draft preparation. WW and HR: data curation. KR and MM: supervision. All authors contributed to the article and approved the submitted version.

## Conflict of Interest

The authors declare that the research was conducted in the absence of any commercial or financial relationships that could be construed as a potential conflict of interest.

## Publisher's Note

All claims expressed in this article are solely those of the authors and do not necessarily represent those of their affiliated organizations, or those of the publisher, the editors and the reviewers. Any product that may be evaluated in this article, or claim that may be made by its manufacturer, is not guaranteed or endorsed by the publisher.
